# Pre-diagnosis quality of life (QoL) in patients with hematuria: comparison of bladder cancer with other causes

**DOI:** 10.1007/s11136-012-0163-1

**Published:** 2012-03-30

**Authors:** Catharina A. Goossens-Laan, Paul J. M. Kil, J. L. H. Ruud Bosch, Jolanda De Vries

**Affiliations:** 1 Department of Urology, University Medical Centre Utrecht, HP:C04.236, PO Box 85500, 3508 GA Utrecht, The Netherlands; 2 Department of Urology, St. Elisabeth Hospital, Tilburg, The Netherlands; 3 Department of Medical Psychology, St. Elisabeth Hospital, Tilburg, The Netherlands; 4 CORPS, Tilburg University, Tilburg, The Netherlands

**Keywords:** Quality of life, Health status, Hematuria, Bladder cancer, Erectile dysfunction

## Abstract

**Purpose:**

To examine quality of life (QoL), health status, sexual function, and anxiety in patients with primary hematuria who later appear to have bladder cancer (BC) and patients with other diagnoses.

**Methods:**

From July 2007 to July 2010, 598 patients with primary hematuria were enrolled in this prospective, multicenter study. Questionnaires (WHOQOL-BREF, SF-12, IIEF, STAI-10-item Trait) were completed before cystoscopy. Diagnosis was subsequently derived from medical files. BC patients were compared with patients with other causes of hematuria.

**Results:**

Cancer was diagnosed in 131 patients (21.9 %), including 102 patients (17.1 %) with BC. No differences were found in the WHOQOL-BREF versus SF-12 psychological or physical health domains. The erectile function was significantly worse in the BC group (9.3 vs. 14.6 for OC, *p* = 0.02). Patients with muscle-invasive BC (MIBC) had the lowest percentage anxious personalities of all BC patients (*p* = 0.04).

**Conclusions:**

Cancer was found in 21.9 % of the patients with hematuria. Pre-diagnosis patients with BC have comparable QoL and HS to patients with OC. Erectile dysfunction was highest in patients with BC. MIBC patients had the lowest percentage anxious personalities of the patients with BC.

## Introduction

Bladder cancer (BC) is the 7th most common cancer worldwide. In the Netherlands, invasive BC was the 8th most common cancer in 2008 [1]. Most patients are being diagnosed after presenting with gross or microscopic hematuria [2, 3]. The golden standard for diagnosis of BC is a cystoscopy of the bladder. When (muscle)-invasive bladder cancer (MIBC) is found, the therapy of choice is the radical cystectomy with bilateral pelvic lympadenectomy. Other curative treatment options are interstitial radiotherapy (IRT; e.g., brachytherapy for small solitary clinical stage II tumors) and external beam radiotherapy (EBRT). When a patient is not eligible for any of the above-mentioned therapies due to comorbidity or preference, a non-curative option usually follows: a transurethral resection of the bladder tumor (TURBT) or palliative radiotherapy.

This complex operation is assumed to affect the patient’s quality of life (QoL) and health status (HS), and sexual functioning as it involves major surgery and having an incontinent or continent urinary diversion. As a result, HS and QoL, both patient reported outcomes, in patients with MIBC undergoing cystectomy are a topic of much interest in urologic oncology. A recent review of Somani et al. [4] on QoL with urinary diversion stated that there is an urgent need to establish the important determinants of QoL of this patient group.

In the few existing prospective studies, baseline QoL is assessed just prior to the cystectomy or shortly after surgery. Psychological, HS, and, health-related QoL measures return to or exceed baseline values [5–7]. In none of these studies, the first measurement of QoL is done before diagnosis of BC. However, being diagnosed with cancer may already cause a changed perspective on QoL. A baseline QoL measurement point before diagnosis can give a more correct reflection of patients’ baseline QoL.

QoL is known to be influenced by health [8], culture [9], social-economic status [10], and personality [11, 12]. Especially, the personality factor trait anxiety is associated with QoL. Studies among breast cancer patients have shown that an anxious personality will react differently to having cancer and undergoing major surgery and will experience a lower QoL compared with patients without such a personality [13, 14].

As stated above, hematuria is the most common presenting symptom of BC. The time before cystoscopy for patients with primary hematuria can serve as a good moment for a baseline measurement in QoL-studies on BC, to use in comparison with postoperative measurements.

The aim of this multicenter study was to examine the patient reported outcome measures QoL, HS, and sexual function and dispositional anxiety in patients with BC before diagnosis was known. The comparison group was other patients with primary hematuria before cystoscopy had taken place. In addition, the diagnoses for primary hematuria were examined.

## Patients and methods

### Patients

All consecutive patients presenting with primary hematuria in one of six academic or large teaching departments of urology (University Medical Centre Utrecht; St. Elisabeth Hospital and Twee Steden Hospital, Tilburg; Onze Lieve Vrouwe Gasthuis, Amsterdam; Jeroen Bosch Hospital, ‘s Hertogenbosch; Catharina Hospital, Eindhoven) in the Netherlands, between July 2007 and July 2010, were eligible for this study. Exclusion criteria were age younger than 18 years, a presumed life expectancy of less than 2 years, dementia, psychiatric disorders or insufficient comprehension of the Dutch language.

**Fig. 1:**
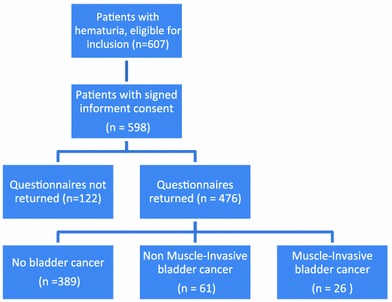
Flowchart study population

Evaluation of the hematuria consisted of history and examination, urinalysis, and cytology. In addition, most patients underwent CT-IVP/urography or ultrasound and/or X-IVP depending on the preference of the individual urologist. Patients who underwent cystoscopy for evaluation of gross or microscopic hematuria were included and asked to complete a set of questionnaires on demographic features, QoL, HS, sexual functioning, and trait anxiety. Five hundred and ninety-eight patients (98.5 %) gave informed consent and were asked to completed the questionnaires before undergoing a cystoscopy or radiological diagnostics, that is, before the diagnosis was known (Fig. 1). Reasons given for not participating or late exclusion (after informed consent was given) were ‘questions too personal’, ‘not interested in participating after reading the questionnaire’, questionnaire not completed before diagnosis. The study was approved by the local ethics committees. All patients provided written informed consent.

### Questionnaires

#### World Health Organization Quality of Life-BREF Questionnaire (WHOQOL-BREF) [15, 16]

This questionnaire is the abbreviated version of the WHOQOL-100 and consists of 26 items covering four domains (Physical, Psychological, Social Relationships, and Environment) and a global QoL and general health facet. Patients also completed the three items from the facet sexual activity from the WHOQOL-100 that are not part of the WHOQOL-Bref. The reliability and validity are satisfactory [17], and the sensitivity to change is good [18].

#### RAND Medical Outcomes Study Short Form-12 (SF-12, version 2) [19]

A 12-item adaptation of the RAND 36-Item Health Survey (SF-36). The SF-12 quantifies health status into two composite scores, the Physical Component Summary (PCS) and Mental Component Summary (MCS) scales. In addition, the SF-12 quantifies health status into eight subscales, including physical functioning, emotional well-being, general health, pain, energy, social functioning, and role limitations due to physical or emotional problems. The summary scales are scored from 0 to 100 and converted to a standardized scale with a population mean of 50 and a standard deviation of 10. A higher score implies a better health status. The reliability and validity are satisfactory [19, 20].

#### State-Trait Anxiety Inventory-Trait scale short form (STAI-10-item Trait) [21]

The STAI was originally developed to investigate anxiety phenomena in ‘normal’ adults but has also proven useful in medical and surgical patients. Trait anxiety concerns differences in individuals in the disposition to respond to stressful situations with varying amounts of stress. The 10-item trait scale (10 statements) asks people to describe how they generally feel. A trait anxiety score of more than 21 was considered high. The psychometric characteristics of this questionnaire are well established and considered good [22].

#### International index of erectile function (IIEF) [23]

The IIEF is a validated, self-administered questionnaire that assesses overall sexual function. It is divided into the 5 domains of erectile function, intercourse satisfaction, orgasmic function, sexual desire, and overall satisfaction. The IIEF demonstrates the sensitivity and specificity for detecting treatment-related changes in patients with erectile dysfunction [23]. After the first year of the study, it became apparent that a significant part of the patients did not participate in the study because of this questionnaire with its sexually related questions. Therefore, from September 2009 onwards new patients did not receive the IIEF questionnaire.

#### Demographic Questionnaire

Patients were asked to answer questions concerning age, marital status, education, and work to evaluate social-economic status.

### Medical records

Data concerning medical diagnosis, tumor stage and grade after pathological examination, and treatment were obtained from the medical records of the patients with informed consent**.**

### Statistical procedure

Independent sample *t* tests and chi-square tests were used to compare: (1) the participants and non-participants and (2) to examine QoL and HS differences between the two patient groups (bladder cancer vs. other causes of hematuria). Correction for covariates age and gender was done with anova univariate analysis. Patients with other forms of cancer were excluded from analyses. When comparing muscle-invasive versus non-muscle-invasive bladder cancer (NMIBC) versus other causes of hematuria with regard to trait anxiety, one way ANOVA was used. Analyzing MIBC to NMIBC group on high anxiety scores, independent sample *t* test was used. Analyses were performed with the Statistical Package for Social Sciences (SPSS version 15.0).

## Results

Four hundred and seventy-six patients (79.6 %) with hematuria participated in this study and answered the questionnaire (Fig. 1). Demographics and clinical features of the participants and non-participants are shown in Table 1. Participants and non-participants only differed with regard to age, with participants being older. Diagnoses of all patients (participants and non-participants) are shown in Table 2. In 226 patients (37.8 %), no pathological finding was detected. Cancer was diagnosed in 131 of the 598 patients (21.9 %), including 102 patients (17.1 %) with BC. NMIBC was found in 71 patients (11.9 %) and MIBC in 31 patients (5.2 %).

**Table 1:** Demographics and clinical features of the participants and non-participants

	Participants (*n* = 476)	Non-participants (*n* = 122)	*p* value
Mean age (years ± SD)	62.5 (11.8)	59.5 (15)	**0.05**
Gender (male)	300 (63)	76 (62.3)	0.6
Diagnosis Benign/malignant	353 (74.2)/110 (23.1)/13 (2.7)	101 (82.8)/21 (17.2)	0.16
Diagnosis bladder cancer	87 (18.3)	15 (12.3)	0.12
NMIBC^a^	61 (70.1)	10 (66.7)	0.13
MIBC^a^	26 (29.9)	5 (41)	0.51
Cystectomy	18 (69.2)	3 (20)	0.44
EBRT	5 (19.2)		
IRT(Brachytherapy)	3 (11.5)		
Nationality (Dutch; missing)	450 (94.5)/10 (2.1)		
Partner: yes/no/missing	378 (79.4)/77 (16.2)/21 (4.4)		
Children: yes/no/missing	393 (82.6)/71 (14.9)/12 (2.5)		
Education: low/high/missing	265 (55.7)/189 (39.7)/22 (4.6)		
Paid work: yes/no/missing	176 (37)/265 (55.7)/35 (7.3)		
Medication: yes/no/missing	345 (72.5)/118 (24.8)/13(2.7)		

*(N)MIBC* (non-) muscle-invasive cancer, *EBRT* external beam radiotherapy, *IRT* interstitial radiotherapy

Besides for age, percentages are between brackets

^a^Percentages of bladder cancer patients

*p* values in bold are significant

**Table 2:** Causes of primary hematuria

Cause	Frequency (*n*)	Percent
None	226	37.8
Prostatic bleeding (BPH; chronic prostatitis)	67	11.2
Calculi (renal/ureteral)	32	5.3
Calculi (bladder)	10	1.7
Cystitis/inflammation (including acute prostatitis)	85	14.2
Endometriosis	2	0.3
Use of oral anticoagulation	8	1.3
Radiation cystitis	2	0.3
Renal cyst with bleeding	3	0.5
Urethral or meatal pathology	10	1.7
Crohn’s disease	1	0.2
Bladder wall-necrosis	1	0.2
Nephrogenic cause	7	1.2
Lost to follow-up	13	2.2
Renal cancer	12	2.0
Ureteral malignancy	5	0.8
Colon cancer	3	0.5
Ovarium cancer	1	0.2
Endometrium cancer	2	0.3
Prostate cancer	6	1.0
Bladder cancer	102	17.1
Total	598	100

Of the participants, 110 patients had cancer (23.1 %) and 87 patients had BC (18.3 %). Only one patient under the age of 50 was diagnosed with BC. Most patients with BC (69.6 %) were older than 60 years. Among the elderly (>75+ years), 23.5 % had BC. Of the patients with BC, 78.4 % were men. Demographics and clinical features of the patients with bladder cancer and with other causes are shown in Table 3.

**Table 3:** Demographics and clinical features of the patients with bladder cancer and other causes

	Bladder cancer	Other causes	*p* value
Mean age (years ± SD)	66.3 (9.5)	61.3 (12.3)	**0.00**
Gender (male)	68 (77.9)	220 (62.9)	**0.01**
Nationality (Dutch)	83 (95.4)	335 (94.9)	0.62
Partner	70 (80)	284 (81)	0.33
Children	69 (79.3)	294 (83.5)	0.12
Education	27 (31)	148 (42)	0.17
Paid work	28 (31)	138 (39.2)	0.46
Medication	59 (67.8)	264 (74.8)	0.37

Besides for age, percentages are between brackets

*p* values in bold are significant

### Patient reported outcomes

Patients’ scores on QoL, health status, sexual function, and anxiety are shown in Table 4.

**Table 4:** Scores for quality of life, health status, sexual function, and anxiety separately for patients with bladder cancer versus patients with other non-malignant causes for hematuria

	Bladder cancer (*n* = 87)	Other causes (*n* = 353)	*p* value with co-variate model age and gender
WHOQOL-BREF			
Overall QoL and general health	3.8 (0.8) [87]	3.7 (0.8) [353]	0.31
Physical health (domain 1)	14.8 (2.8) [87]	14.5 (3.0) [350]	0.56
Psychological health (domain 2)	14.9 (2.1) [87]	14.8 (2.3) [352]	0.73
Social relationships (domain 3)	14.0 (3.4) [87]	14.4 (3.2) [351]	0.77
Environment (domain 4)	15.7 (2.2) [87]	15.8 (2.4) [351]	0.76
Sexual satisfaction	11.1 (4.4) [87]	12.1 (4.7) [351]	0.34
SF-12			
General health perceptions	46.6 (19.9) [87]	45.7 (20.3) [352]	0.70
Physical functioning	73 (30) [87]	74.4 (31) [351]	0.96
Social functioning	19.1 (18.3) [87]	19.8 (19.4) [346]	0.90
Role limitations physical	66.7 (46.2) [87]	60.5 (46.2) [351]	0.44
Role limitations emotional	90.3 (38.8) [87]	81.7 (35.2) [346]	0.10
General mental health	72.9 (16.8) [87]	70.5 (18.6) [347]	0.53
Energy/fatigue	60.7 (25.2) [87]	56.4 (25.3) [347]	0.39
Bodily pain	76.2 (22.9) [87]	75.5 (23.6) [345]	0.93
Physical component scale	65.6 (22.7) [87]	64.5 (24.9) [343]	0.80
Mental component scale	60.7 (14.9) [87]	57.2 (14.5) [342]	0.15
IIEF			
Erectile function	9.3 (8.1) [29]	14.6 (9.2) [80]	**0.02**
Patients with ED (cutoff 25 points)*	93.1 %	78.8 %	
Intercourse satisfaction	4.1 (5.1) [32]	6.8 (5.9) [87]	0.07
Orgasmic function	3.4 (3.9) [31]	5.3 (4.2) [89]	**0.05**
Sexual desire	4.5 (2.0) [29]	5.1 (2.1) [89]	0.26
Overall satisfaction	5.1 (2.5) [25]	6.2 (2.5) [81]	0.17
Trait anxiety	16.9 (4.9) [85]	17.6 [340]	0.29
High score on anxiety (score ≥22)	16.5 %	24.7 %	0.15

*ED* erectile dysfunction

Patients with other forms of cancer were excluded from analyses

Scores are represented in means. SD are between brackets

Number of questionnaires with the item completed between square brackets

*p* values in bold are significant

* Erectile dysfunction with cut-off point at 25 points

No significant differences were found for general QoL and the four QoL domains. The SF-12 showed no differences on HS between patients with BC versus patients with OC.

The IIEF showed a significant effect on erectile function (*p* = 0.02) and orgasmic function (*p* = 0.05). OC patients had better scores than BC. In line with this finding, erectile dysfunction was highest among patients with BC (93 % vs. OC 79 %).

The mean score of trait anxiety indicated normal anxiety in both groups (BC and OC). Patients with MIBC had significantly lower scores on trait anxiety compared with the other BC patients (*F* = 4.94, *p* = 0.03), only 7 % of all patients with MIBC had a high anxious personality in comparison with 20 % in the NMIBC group and 25 % of all patients (*x* ^2^ = 1.18, *p* = 0.05).

## Discussion

We examined the QoL, HS, sexual functioning, and dispositional anxiety in patients with BC before diagnosis was known. The comparison group was other patients with primary hematuria before cystoscopy had taken place.

### Quality of life and health status

In the urologic oncology community, there is no standardized assessment protocol for QoL-studies, and a wide variation exists in QoL-outcome studies [24]. A major limitation is that the few prospective studies all report a “baseline” assessment of HS and/or QoL that is done, only after MIBC is confirmed. However, the diagnosis cancer by itself is an almost certain reason for a change in QoL. It was our aim to assess QoL and HS before diagnosis in order to get a good baseline assessment. Therefore, we asked all patients with primary hematuria to complete questionnaires on QoL, HS, sexual function, and level of trait anxiety before a diagnosis was established. QoL-studies for MIBC mostly involve only HS which is not equivalent to QoL [7, 25]. HS indicates where there are limitations in physical functioning as impact of the disease, whereas QoL also reflects to what extent the patients are bothered by these limitations in daily life [14, 26].

The current study shows that the patients with unknown diagnosis of BC appeared to have the same baseline results on QoL and HS in comparison with the patients with other diagnosis. The BC patients perceived their sexual functioning as lower, and they also had the lowest percentage of anxious personalities.

Erectile dysfunction was highest (93.1 %) and orgasmic function lowest among patients with BC compared with the patients with other diagnoses. The underlying disease, although not known by the patient, seems to have an impact on his/her sexual life. Although it is known that sexual function decreases after the cystectomy, the fact that patients already have a decrease in function before diagnosis is new information [7, 27]. A history of smoking is a known risk factor for BC, and the fact that smoking can give rise to cardiovascular disease that is an important cause of erectile dysfunction could be an explanation for our finding.

Furthermore, patients with BC had the lowest percentage of anxious personalities (17 %), especially patients with MIBC (7 %). This may be a reason for having a higher-stage disease at presentation in the hospital as their anxiety level prevents these patients from visiting their doctor when symptoms first emerge. As this finding is based on only 26 versus 61 patients with a significance of *p* < 0.03, further research with more power is needed for confirmation.

Our study had strengths and limitations that merit comment.

This prospective study is multicenter. To our knowledge, this is the first study to investigate QoL in patients with hematuria and to investigate pre-diagnosis QoL in patients with bladder cancer. The best population to study this research question would be the general population. However, in the Netherlands in 2009, there were 5,100 cases of newly diagnosed bladder cancer, with a lifetime cumulative risk of 2 % in a population of 16 million people [1]. This low risk makes screening the general population for the current research question unfeasible. As hematuria is the key symptom of bladder cancer, the population presenting with hematuria could be considered second best.

A limitation is the early withdrawal of the IIEF questionnaire. Particularly as the results of the patients who did complete the IIEF showed a difference in sexual function. Despite oral and written information about this questionnaire to patients when informing patients about the study, the proportion of patients refusing participation due to the IIEF made us decide to withdraw the list. Further limitations are that no difference is made during the inclusion between the two forms of hematuria, and the patients’ smoking history is unknown.

There are multiple studies on QoL after cystectomy [7, 25]. Most studies only use HRQoL questionnaires and do not compare QoL and HS in the same study population. No study has as yet addressed the issue of a baseline measurement with an unknown diagnosis, as in our study. Our study shows that pre-diagnosis QoL and HS do not differ between patients with and without bladder cancer. A follow-up study is initiated to evaluate QoL after cystectomy for BC using this baseline measurement with an unknown diagnosis and to see how QoL is affected by the surgery.

In conclusion, cancer was found in 21.9 % of the patients with hematuria.

Pre-diagnosis patients with BC have comparable QoL and HS to patients with OC. Erectile dysfunction was highest in patients with BC. Patients with MIBC had the lowest percentage of anxious personalities.

## Abbreviations

BC: Bladder cancer
CT-IVP/CT-urography: Computer tomography—intravenous pyelography
EBRT: External beam radiotherapy
HRQoL: Health related quality of life
HS: Health status
IIEF: International index of erectile function
IRT: Interstitial radiotherapy
(N)MIBC: ((non-) Muscle-invasive bladder cancer)
OC: Other causes
QoL: Qualtiy of life
SF-12: RAND Medical Outcomes Study Short Form-12
SPSS: Statistical package for social sciences
STAI: State-Trait Anxiety Inventory-Trait scale short form
X-IVP: Rontgen intravenous pyelograpy
WHOQOL-BREF: World Health Organization Quality of Life-BREF Questionnaire
